# Posterior dynamic stabilization in the lumbar spine – 24 months results of a prospective clinical and radiological study with an interspinous distraction device

**DOI:** 10.1186/s12891-016-0945-7

**Published:** 2016-02-18

**Authors:** Dorothea Daentzer, Christof Hurschler, Frank Seehaus, Christine Noll, Michael Schwarze

**Affiliations:** Department of Orthopedics, Spine section, Hannover Medical School, Diakovere Annastift gGmbH, Anna-von-Borries-Str. 1-7, D-30625 Hannover, Germany; Laboratory for Biomechanics and Biomaterials, Hannover Medical School, Anna-von-Borries-Str. 1-7, 30625 Hannover, Germany; CeramTec GmbH, Luitpoldstr. 15, 91207 Lauf, Germany

**Keywords:** Interspinous distraction device, Lumbar spine, Posterior dynamic stabilization, Roentgen stereophotogrammetry, Wallis implant, Wallis spacer

## Abstract

**Background:**

Interspinous distraction devices (IDD) are due to maintain or restore intersegmental range of motion (iROM) in a controlled fashion with the aim of stabilization the affected level dynamically. The following study is the first to present clinical and radiological data with the Wallis® spacer during a follow-up of 24 months.

**Methods:**

Ten patients underwent posterior dynamic stabilization (PDS) of the lumbar spine with an IDD (Wallis® spacer) and were controlled clinically and radiologically after 3, 6, 12, and 24 months in a prospective study design. Pain intensity, functional disability and life quality were assessed by use of subjective scores. Motion analyses were performed with the help of lateral functional x-rays to determine the iROM of the operated segments and total ROM (tROM) of the lumbar spine. In addition, roentgen stereophotogrammetric analysis (RSA) was used to measure the iROM of the treated levels.

**Results:**

During the postoperative course pain and disability most clinical scores were significantly improved. After 24 months we observed statistically significant reduction in back pain intensity with a mean value of 6.0 on visual analog scale (VAS) before surgery and of 2.7 at the latest evaluation. The leg pain was also decreased without statistical significance from 4.7 preoperatively to 2.1 at final follow-up. The functional disability according to Oswestry Disability Index (ODI) and Roland-Morris Disability Questionnaire (RM) was decreased both with statistical significance at all examination dates with a mean value in ODI of 40.0 % before operation and of 17.3 % after 2 years and an initial mean value in RM of 55.2 and of 23.5 % after latest follow-up. After 24 months, the results of the health related quality of life score also showed much better values with only two exceptions. The iROM of the treated levels was reduced during each follow-up examination with preserved residual mobility. Directly postoperatively and after 3 and 12 months intersegmental mobility was statistically significantly decreased with an average iROM of 6.62° before operation and of 2.69° few days after surgery, of 3.79° and 3.16° 3 and 12 months later. At 6 (4.37°) and 24 (4.01°) months follow-up iROM was also but not statistically significantly reduced. The mean tROM did not change significantly during all postoperative controls.

**Conclusions:**

The radiological findings support the thesis of posterior dynamic stabilization by the used implant. The positive clinical findings should be interpreted with caution because of the limited number of patients and the missing control group.

## Background

Interspinous distraction devices (IDD) are fixed between two adjacent spinous processes of the lumbar spine and are intended to maintain or restore segmental motion while avoiding disadvantages of rigid spinal fusion [1]. Therefore, they control intervertebral motion and act as a posterior dynamic stabilization (PDS) system. However, indication for IDD are still under discussion to date. Some authors use solely IDD [2, 3], while additional use of IDD after decompressive procedures to prevent instability and to keep the operated level in a rather flexed position to maintain the spinal canal and neuroforamen open is more commonly applied [4–8]. Furthermore, IDD are assumed to unload and to protect the facet joints and to avoid accelerated adjacent-segment degeneration [1].

The first IDD device certified for clinical use is the “Wallis® spacer” [9]. Long-term results have been published by its developer Sénégas et al. but without a control group [10]. The aim of the following publication is to show the postoperative course continuously during a follow-up period of 24 months and to assess pain intensity, functional disability and health related quality of life. Furthermore, intersegmental range of motion (iROM) and total ROM (tROM) of the lumbar spine were analyzed by the use of conventional functional x-ray imaging in addition to roentgen stereophotogrammetric analysis (RSA) [11]. We were thus able to determine iROM during various activities and also to evaluate the remaining segment mobility after treatment with different surgical techniques such as fusion or arthroplasty [12–17].

To the authors’ knowledge, no investigation on PDS was conducted with a high-accuracy method such as RSA to date. Therefore, in this study the radiological data including RSA is to demonstrate the in vivo mobility after implantation of an IDD (Wallis® spacer).

## Methods

Ten patients (seven women and three men, mean age 64.4 years) were included in this prospective single-centre study which was approved by the Institutional Review Board (Hannover Medical School No. 4809) after biometrical power calculation of number of cases. All participants provided consent. Inclusion criteria were therapy resistant or progressive back and/or leg pain under conservative treatment due to spinal canal stenosis with (*n* = 3) or without disc prolapse (*n* = 4), slight degenerative spondylolisthesis (*n* = 2, in one person with spinal canal stenosis) and facet joint arthrosis (*n* = 1). Exclusion criteria were spondylolisthesis more than grade one, segmental scoliosis, trauma, tumor, infection and osteoporosis which was excluded by Dual-X-Ray-Absorptiometry. Eight patients had a typical neurogenic intermittent claudication. The most affected level was L4/5 in nine cases, one person was treated in L2/3. We used the iROM and tROM as a surrogate metric for spine stability.

### Implant and operation

The implant (Wallis® spacer, Zimmer Spine SAS, Bordeaux, France) was inserted between two neighboring vertebral arches and additionally fixed with two tension bands of polyester which were wrapped around both adjacent spinous processes. Eight patients also had decompressive surgery with (*n* = 3) or without (*n* = 5) removal of a disc prolapse.

For RSA, three to five tantalum markers with a diameter of 1 mm were inserted in the posterior bony structures of each adjacent vertebra (lamina, articular process, spinous process).

### Clinical evaluation

All patients filled out a questionnaire with assessment of their intensity for back and leg pain by the visual analog scale (VAS), of their functional impairment by the Oswestry Disability Index (ODI) and the Roland-Morris Disability Questionnaire (RM) and of their health related quality of life by the Short-Form-36 Health Survey (SF-36) directly after inclusion into the study before operation and at further follow-up dates after 3, 6, 12, and 24 months. Furthermore, walking distance was documented and all persons had clinical and neurological examination during each control by the same examiner.

### Radiological analysis and RSA

**Fig. 1:**
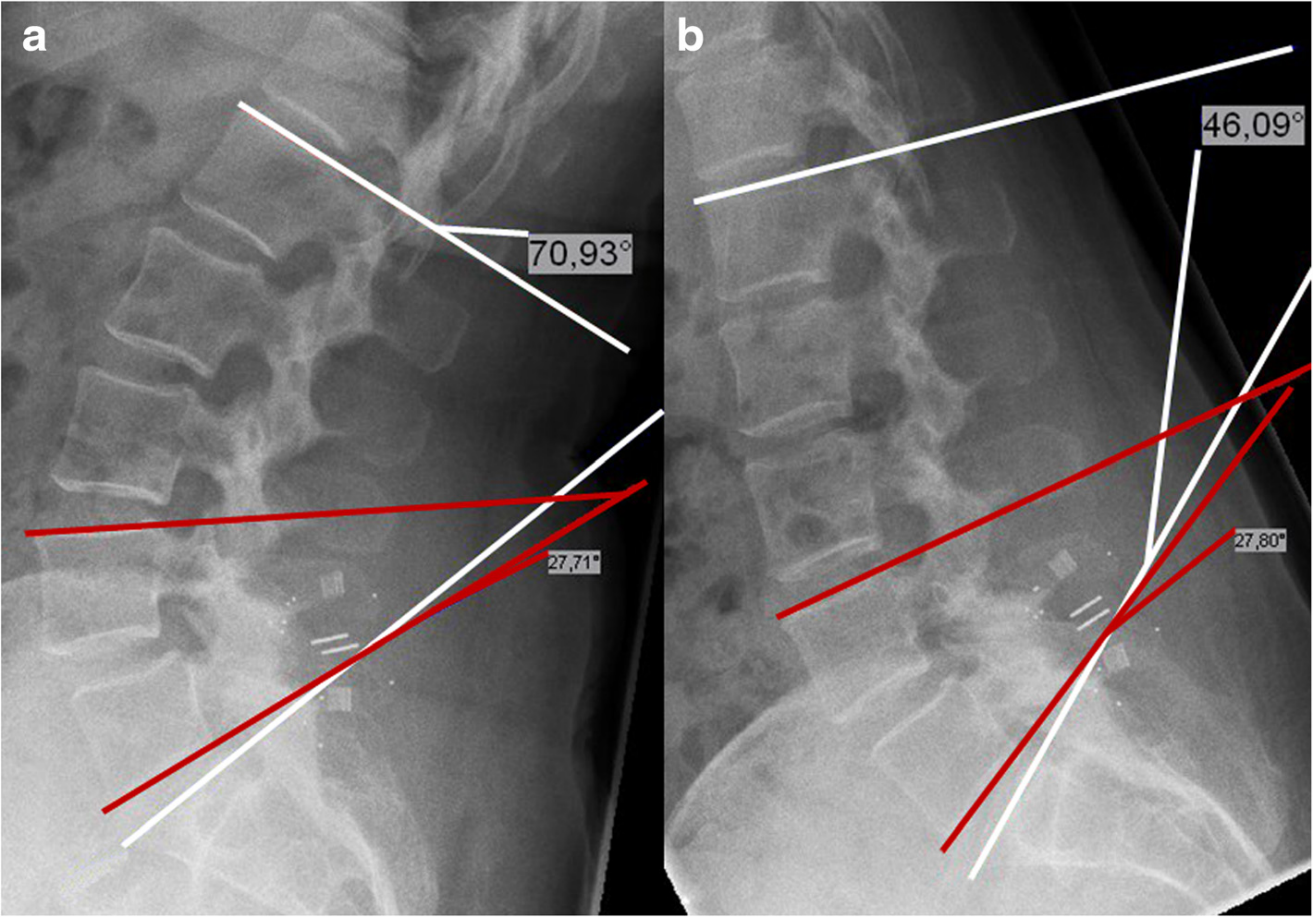
Measurement of the segmental and total lumbar angle. Measurement in the lateral roentgenogram was performed with the Cobb method with the implant fixed between the spinous processes of L4 and L5. The red lines show the segmental angle measured between the upper endplate of L4 and the lower endplate of L5, the white lines label the total lumbar angle measured between the upper endplate of L1 and the endplate of S1. **a**: Extension, **b**: Flexion

Conventional functional x-rays of the lumbar spine were performed in a standardized manner pre- and postoperatively (between 3 and 10 days), as well as at each follow-up date. These images were analysed in regard to the tROM of the lumbar spine by measuring the angle between the first lumbar vertebra (upper endplate of L1) and the endplate of the sacrum (S1) and then calculating the difference between the extension and flexion images. The iROM between the upper and lower vertebrae of the operated segments was calculated building the difference of the intervertebral angles in extension and flexion using the Cobb method (Fig. 1).

For RSA, radiographs were taken up to ten days after surgery and at 3, 6, 12, and 24 months post-op in a uni-planar setup using a carbon-fiber calibration box (box10, Medis specials). The angle between the x-ray paths was 40 deg. X-ray tubes (Digital Diagnost, Philips) exposed standard photostimulated luminescence plates with the dimension of 350 × 430 mm without the use of scatter grids. The plates were digitized resulting in an eight bit gray-scale image with a resolution of 125 dpi. X-ray cathode voltage was 125 kV and time-current was 40 mAs. No double examinations were conducted to minimize x-ray exposure of the patients. Persons were positioned in standardized extension and flexion position lying on the right side by an experienced examiner [18]. They lay on a flat table with the calibration box directly under the examined area of interest. Spinal segment motion was calculated using the MBRSA software (Version 3.31, Medis specials) with a standard protocol and a single examiner. The markers in the upper and lower vertebrae constituted the rigid bodies. Rigid body error threshold was 0.50 mm, with one exception at a single follow-up where 0.57 mm was required. The lower rigid body was used as reference, with the coordinate system aligned to the calibration box. Rotations around the z-axis (perpendicular to the image plane) were calculated, whereas positive rotation corresponds to flexion.

### Statistical analysis

For statistical analysis of all data the *t*-test for related samples with a significance level of *p* < 0.05 was chosen to investigate differences at follow-up dates compared with the preoperative values.

## Results

### Clinical results

The intraoperative course was uneventful in all ten patients. Only one woman needed follow-up surgery because of a wound healing problem without an infection. None of the patients had postoperative neurological complications. One male patient was excluded from the study within the first 3 months because of conversion to fusion surgery due to persisting complaints. The follow-up data of the remaining nine patients are presented here.

### Walking distance

Before surgery, the walking distance was reduced in eight patients to between 10 and 2000 m with a mean of 182 m. After 24 months, five patients had no more restrictions in walking. In the other four persons, the average walking distance had increased to at least 250 m and up to 2000 m (mean 1563 m).

### Clinical outcome

**Fig. 2:**
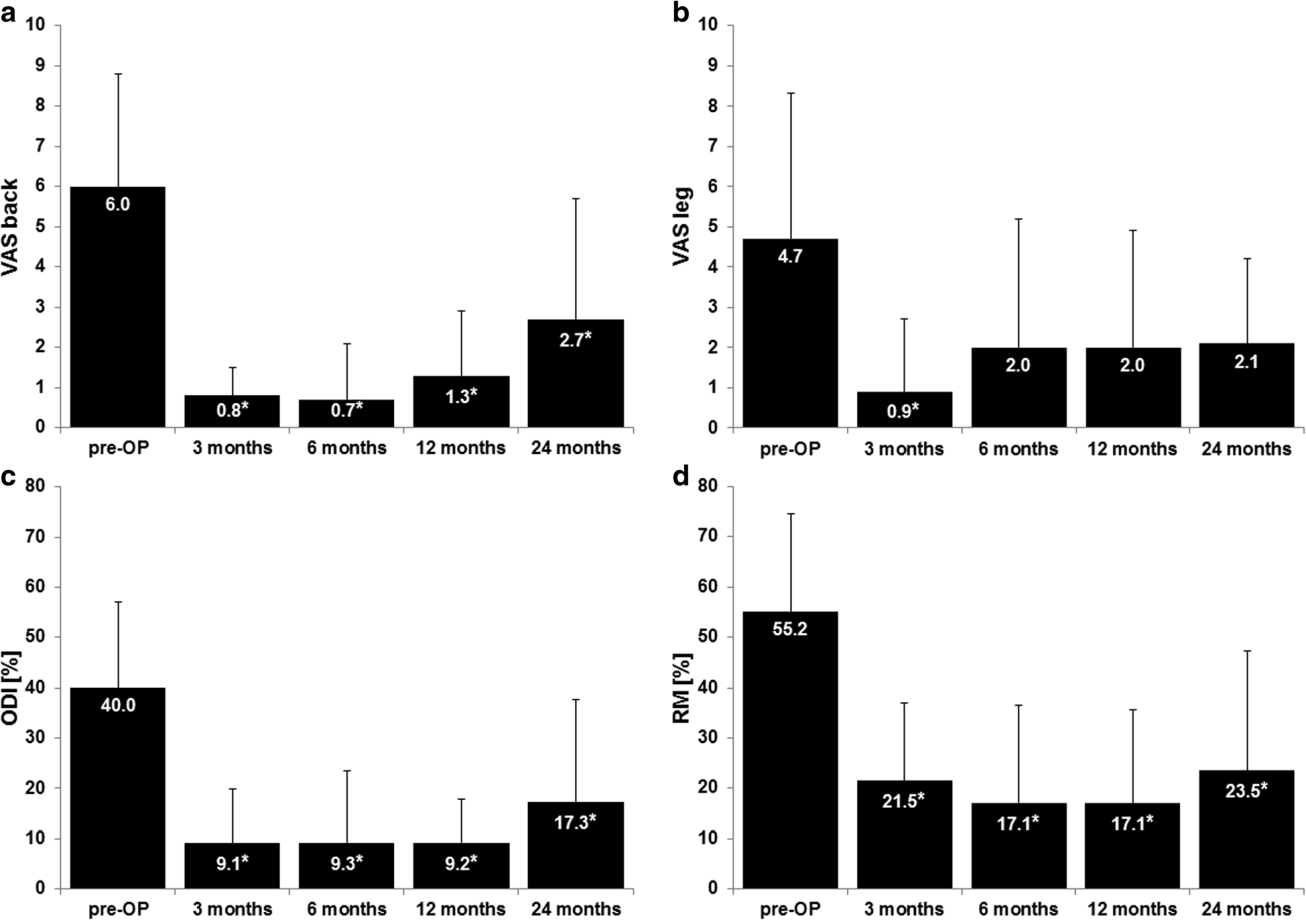
Results of the scores. Data were presented for pain intensity by VAS for back (**a**) and for leg (**b**) and for functional impairment by ODI (**c**) and RM (**d**); pre-OP means preoperatively, *indicates statistical significant difference to the preoperative value

A statistically significant decrease in back pain and functional disability (ODI and RM) was observed for all patients at every follow-up interval (Fig. 2, Table 1). Patients also showed reduced leg pain, which was however statistically significant only at 3 months follow-up.

**Table 1:** Pain intensity for back and leg and functional disability

Time	VAS back		VAS leg	
	mean ± SD	*p*-value	mean ± SD	*p*-value
Pre-OP	6.0 ± 2.8		4.7 ± 3.6	
3 months	0.8 ± 0.7	0.000*	0.9 ± 1.8	0.009*
6 months	0.7 ± 1.4	0.001*	2.0 ± 3.2	0.059
12 months	1.3 ± 1.6	0.004*	2.0 ± 2.9	0.058
24 months	2.7 ± 3.0	0.042*	2.1 ± 2.1	0.060
	ODI		RM	
	mean ± SD	*p*-value	mean ± SD	*p*-value
Pre-OP	40.0 ± 17.1		55.2 ± 19.4	
3 months	9.1 ± 10.7	0.012*	21.5 ± 15.5	0.009*
6 months	9.3 ± 14.2	0.002*	17.1 ± 19.5	0.005*
12 months	9.2 ± 8.6	0.002*	17.1 ± 18.5	0.002*
24 months	17.3 ± 20.3	0.017*	23.5 ± 23.9	0.006*

Mean values for VAS back and leg and for ODI and RM; *p*-values are referred to preoperative value; *SD* standard deviation, *pre-OP* preoperatively *shows statistical significant differences between follow-up and preoperative data with *p*-value less than 0.05

**Fig. 3:**
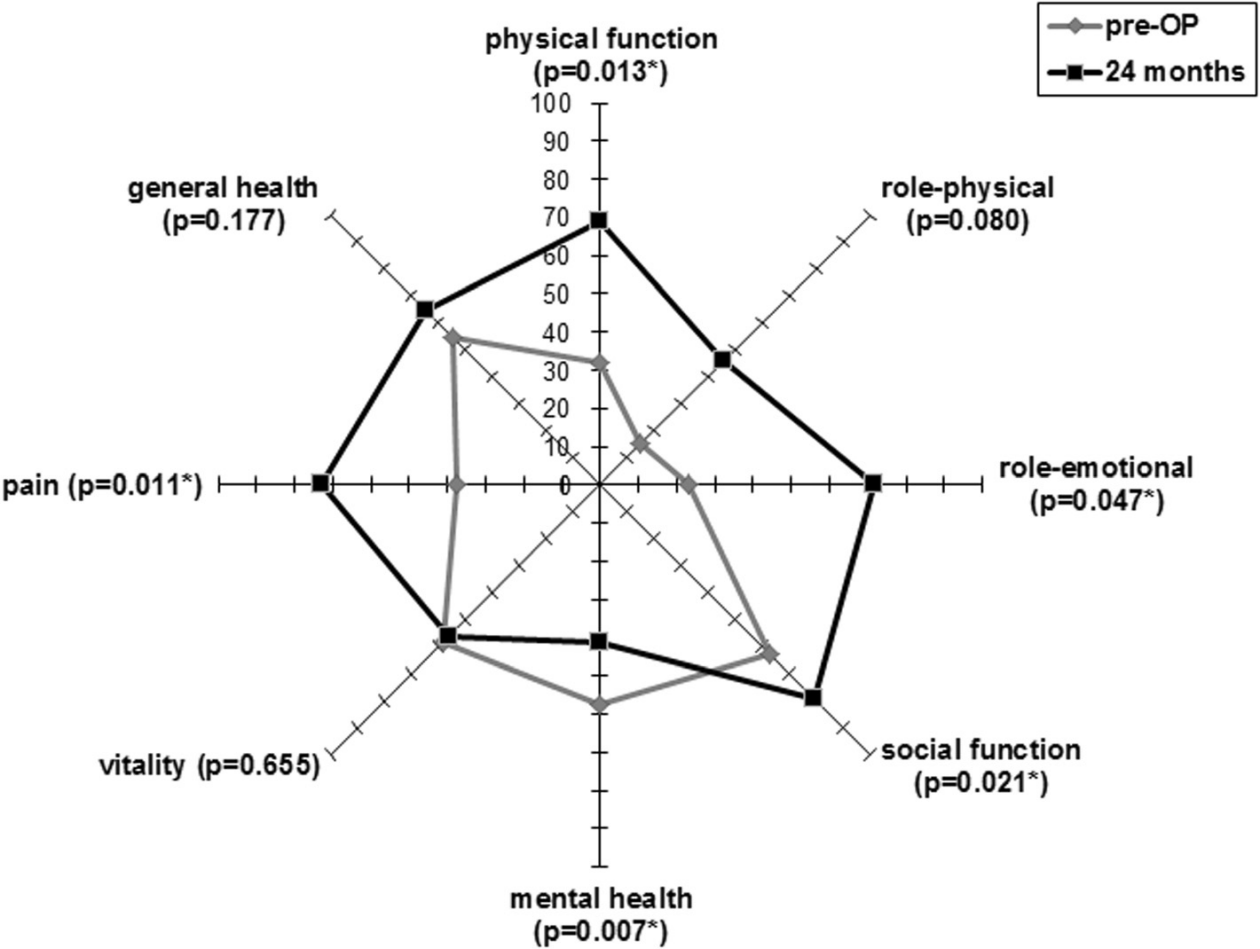
SF-36 before operation and 24 months later. pre-OP means preoperatively, *indicates statistical significant difference to the preoperative value

The data from the SF-36 were improved in six of the eight items (all but for mental health and vitality) with statistical significant differences with regard to physical function, role-emotional, social function and pain (Fig. 3).

### Radiological results and RSA

**Fig. 4:**
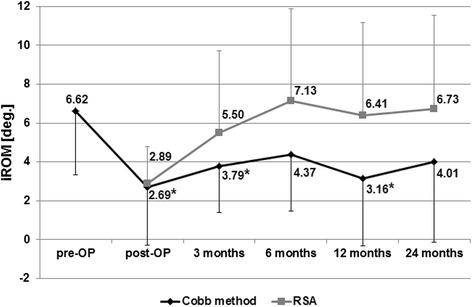
Development of the iROM in degree (deg.) to the different time points. pre-OP means preoperatively, post-OP means soon after surgery, measured by the Cobb method and RSA, *indicates statistical significant difference to the initial angle

Before surgery, iROM of the operated segments measured by Cobb’s method was 6.62° ± 3.30° (Fig. 4, Table 2). Directly after operation it was decreased to 2.69° ± 2.96° with inconstant increasing during the further course to 3.79° ± 2.38° after 3 months, 4.37° ± 2.88° after 6 months, 3.16° ± 3.48° after 12 months and 4.01° ± 4.15° after 24 months.

**Table 2:** iROM and tROM during the postoperative course

Time	iROM, mean ± SD (deg.)			tROM, mean ± SD (deg.)	
	Cobb method	*p*-value	RSA	Cobb method	*p*-value
Pre-OP	6.62 ± 3.30		-	26.01 ± 10.29	
Post-OP	2.69 ± 2.96	0.010*	2.89 ± 1.89	19.65 ± 5.67	0.056
3 months	3.79 ± 2.38	0.034*	5.50 ± 4.21	26.63 ± 8.19	0.283
6 months	4.37 ± 2.88	0.161	7.13 ± 4.77	28.35 ± 6.77	0.204
12 months	3.16 ± 3.48	0.040*	6.41 ± 4.75	25.73 ± 7.68	0.399
24 months	4.01 ± 4.15	0.175	6.73 ± 4.82	31.45 ± 7.87	0.051

The ROM-data were calculated from the difference of angles in flexion and extension; *p*-values are referred to preoperative value;*SD* standard deviation, *pre-OP* preoperatively, *post-OP* postoperatively *shows statistical significant differences between follow-up and preoperative data with *p*-value less than 0.05

Segmental ROM of the treated levels calculated with RSA could be determined for the first time directly after surgery and was 2.89° ± 1.89° with inconsistent increase over the follow-up period to 5.50° ± 4.21° after 3 months, 7.80° ± 5.23° after 6 months, 4.90° ± 3.33° after 12 months and 6.73° ± 4.82° after 24 months (Fig. 4, Table 2). As the RSA tantalum markers were not in situ before surgery, we could not compare to the preoperative values.

The discrepancy of the conventionally determined iROM and the intervertebral motion measured by RSA was low with a mean of 2.90° (SD: 2.07), but varied among patients. The quality of the RSA was assured by determining mean rigid body error (0.31 ± 0.49 mm) and condition number (80 ± 21).

**Fig. 5:**
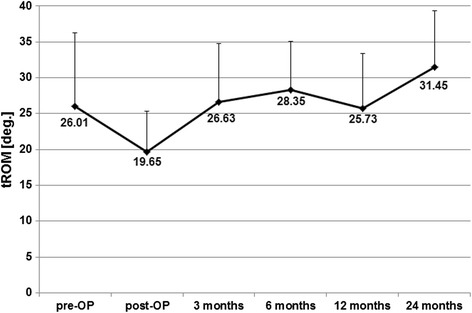
Development of the tROM in degree (deg.) to the different time points. pre-OP means preoperatively, post-OP means soon after surgery, measured by the Cobb method L1 to S1

The tROM before operation was 26.01° ± 10.29° with reduction to 19.65° ± 5.67° some days later. During the whole follow-up we did not observe any statistical significant differences to the initial value (26.63° ± 8.19° after 3 months, 28.35° ± 6.77° after 6 months, 25.73° ± 7.68° after 12 months and 31.45° ± 7.87°) (Fig. 5, Table 2).

## Discussion

In this study we present clinical and radiological findings after PDS with an IDD with the main focus of evaluation of the in vivo intervertebral and total lumbar spine mobility by the use of conventional functional x-ray imaging and with high-accuracy RSA during a follow-up of 24 months.

The analysis of the iROM of the treated segments shows statistical significant reduction directly after operation and after 3 and 12 months with still but not significant decreased ROM after 6 and 24 months when compared to the preoperative value. This course corresponds somewhat to data from literature with stronger decrease of iROM shortly after operation (from 9.28° preoperative to 4.75° postoperative) and slight increase in the following time period (6.65° at last follow-up) which is the only available publication about this topic [2]. However, in the retrospective study by Sobottke et al. the 18 patients with the Wallis® implant had a follow-up of only 7.2 months. These data nevertheless suggest that IDD have the capability to provide dynamic stabilization of the affected levels in the lumbar spine. We also have no reasonable doubt, that this effect could be maintained 24 months and beyond, although data with a follow-up longer than 2 years are still not available.

In interpretation of the iROM data several limitations should always be kept in mind. We have to consider the intra- and interobserver variability up to 8.8° when using the Cobb method [19, 20]. Furthermore, patients show intra-subject variability in spine mobility which is for pre- and postoperative condition, especially after dynamic stabilization. Furthermore, spine mobility depends on the patient’s cooperation during examination and condition with possible restricted mobility in case of pain.

It should be noted, that since RSA relies on the intraoperatively implanted tantalum markers, it can only provide data postsurgically and cannot capture the preoperative referenced ROM. Intervertebral ROM measured with RSA showed a similar tendency to the values of iROM determined by Cobb method with slight but not uniform increase of ROM with increasing follow-up time. The RSA based iROM observed in our study were constantly higher than the values measured conventionally. This is different to a comparative study with patients after lumbar disc replacement by Park et al. who found a mean difference in segmental motion of 2.4° between RSA and digital Cobb technique with lower values for RSA [17]. The overall discrepancy of the conventionally determined iROM and the data measured by RSA was low in our patients (2.90°). While the Cobb method generally has an intra- and interobserver variability up to 8.8° RSA is known to be the most exact method for motion analysis with an accuracy between 0.15° and 1.15° [19, 20]. For clinical decision making, the Cobb method’s accuracy is sufficient, for certain research questions RSA should be applied.

The direct postoperative mobility of the total lumbar spine was clearly but not significantly reduced, maybe because of patient’s discomfort or wound pain a few days after surgery, with a continuous increase during the further time period with one exception after 12 months with again slight decrease. The tROM after 24 months was the highest but without statistical significance, probably due to the relatively small number of cases we investigated.

Our radiological findings concerning iROM correlate well with other biomechanical studies. In an experimental test setup with analysis of four different IDD all implants showed significant and more than 50 % decrease of extension without any stabilizing effect in lateral bending or axial rotation but with strong reduction of intradiscal pressure [21]. Only the Wallis® spacer demonstrated a tendency to restabilize the specimens in flexion nearly to the values to the intact condition. Similar findings were published by Lafage et al. [22], who evaluated iROM of the Wallis® implant in vitro and by finite-element analysis. Mainly reduced flexion-extension ROM without suppressing overall mobility with lowered stress in the disc was found. In another experimental study the Wallis® spacer underwent biomechanical analysis against intact condition and a semi-rigid pedicle-screw based implant [7]. Again the IDD lead to primary stabilizing effect with restriction of motion predominantly in the sagittal plane.

Summarizing our radiological results and the findings of the experimental studies, the effect of IDD can be considered as proven with regards to stabilizing the addressed lumbar segment. In addition to the cited biomechanical studies, we have now observed that the stabilizing influence of the investigated IDD is not only of short-term nature, but has a mid- to long-term effect at least to the minimum of 24 months we were able to follow-up.

We are aware that the clinical results of the presented study should be interpreted with caution because of the monocentric study design without any control group and the small number of patients. However, there is a lack of data from prospective trials dealing with the Wallis® spacer without additional fusion procedures in another level to date. One retrospective study with this device was published by its developer and demonstrated a survivorship of this system of 82.8 % at 10 years and of 75.9 % at 14 years but gave no information regarding radiographic findings [10]. Sobottke et al. retrospectively analyzed 18 persons treated with the same implant without decompression and observed statistical significant pain reduction in the postoperative course [2]. The strength of our clinical trial is the prospective study design with continuous monitoring of pain intensity for back and leg, functional disability and health related quality of life with a follow-up period of 24 months. The data of the scores as well as the development of the walking distance are very promising with almost always significant improvement, but we cannot compare our findings with patients who underwent other surgical techniques such as decompression without an IDD or isolated implantation of IDD without decompression because of the lack of a control group.

Regarding clinical results two systematic reviews were published comparing IDDs with decompressive surgery in patients with spinal canal stenosis [23, 24]. Wu et al. performed a meta-analysis of two randomized controlled trials and three non-randomized prospective comparative studies with 204 patients in the IDD group and 217 persons in the decompression collective [23–29]. Five different devices were investigated (X-STOP, Aperius®, Coflex®, DIAM™, distraXion). Both treatment groups showed mostly significant improvement in clinical outcome scores (VAS for back and leg, ODI and RM). However, after pooled analysis the authors observed no significant difference between IDD and decompression patients. Furthermore, they found a similar complication rate but a significantly higher incidence of reoperations with 19.3 % in the IDD group than in the decompression collective with 6.9 %. Similar results were found by Hong et al. who conducted a meta-analysis with 20 studies including 3155 patients after implantation of an IDD (X-STOP, Aperius®, Coflex®, DIAM™, Wallis®, SPIRE®) and 50,983 patients after decompression [24]. In summary, both surgical procedures led to clinical improvement but without significant difference between the two treatment options for improvement rate, VAS for back and leg or ODI. Again, reoperation rate was higher in IDD group than in decompression group (16.5 % versus 8.7 %).

Radiological findings were mostly not the focus of these studies and were only rarely reported. Thus, in the investigation of Kim et al. the used DIAM™ and Coflex® spacers led to decreased iROM in the affected level directly after operation with increasing during time with values close to the initial data at the last follow-up after an average of 71 months [27]. The reported iROM was always clearly higher than in our study and the differences were not statistically significant.

## Conclusions

According to the radiological results of this study, the used Wallis® implant stabilizes dynamically expressed by mostly significant reduction of intervertebral ROM of the operated lumbar spinal segments. The positive clinical findings should be interpreted with caution because of the small number of patients and the lack of a control group.

### Ethical standards

All procedures have been approved by the local ethics committee and have been performed in accordance with the ethical standards of the institutional and national research committee and with the 1964 Declaration of Helsinki and its later amendments or comparable ethical standards.
